# Association of Physical Activity and Exercise with Physical Performance and Muscle Mass in Older Adults: Results from the Longevity Check-Up (Lookup) 7+ Project

**DOI:** 10.3390/jcm12247521

**Published:** 2023-12-05

**Authors:** Hélio José Coelho-Júnior, Riccardo Calvani, Anna Picca, Matteo Tosato, Francesco Landi, Emanuele Marzetti

**Affiliations:** 1 Department of Geriatrics, Orthopedics and Rheumatology, Università Cattolica del Sacro Cuore, L.go F. Vito 1, 00168 Rome, Italy; francesco.landi@unicatt.it (F.L.); emanuele.marzetti@policlinicogemelli.it (E.M.); 2 Fondazione Policlinico Universitario “A. Gemelli” IRCCS, Largo A. Gemelli 8, 00168 Rome, Italy; picca@lum.it (A.P.); matteo.tosato@policlinicogemelli.it (M.T.); 3 Department of Medicine and Surgery, LUM University, Str. Statale 100 km 18, 70100 Casamassima, Italy

**Keywords:** handgrip strength, chair-stand test, muscle power, strength training, aerobic exercise, body composition

## Abstract

Regular engagement in physical activity (PA) or physical exercise (PE) is effective at improving physical performance and body composition in older adults. Less is known about the benefits that may be obtained through combining PA with PE and whether the effects of activity habits differ between men and women. This study cross-sectionally investigated the association of PA and/or PE with physical performance and anthropometric measures in a large and relatively unselected sample of older adults enrolled in the Longevity Check-up (Lookup) 7+ project. Participants were individuals 65 years and older living in the community who were recruited in unconventional settings across Italy. Adherence to PA or PE was operationalized as involvement in light walking or various types of exercise, respectively, at least twice weekly for a minimum of 30 min per session throughout the last 12 months. Physical performance measures included handgrip strength and five-time sit-to-stand (5STS) tests. Lower-limb muscle power and appendicular skeletal muscle mass (ASM) were estimated through validated equations. We analyzed data of 4119 participants, of whom 2222 (53.4%) were women. The mean age was 72.8 ± 5.8 years in men and 72.1 ± 5.4 years in women. Regular engagement in PA + PE was reported by 139 (7.3%) men and 100 (4.5%) women. Results indicated that regular walking activity and/or PE were significantly associated with better physical performance and greater ASM with sex-specific patterns. Associations were also influenced by the type of activity, physical performance assessment tool, and anthropometric parameters. Men engaged in PA + PE performed better on the 5STS test and had greater handgrip strength, ASM, and relative and specific muscle power than those practicing either PA or PE. In women, the combination of PA and PE was associated with greater handgrip strength. The findings of this study indicate that older adults regularly practicing PA + PE had better physical performance than those who only engaged in either modality. In men, the combination of PA and PE was also associated with greater ASM.

## 1. Introduction

The loss of physical performance is an established biomarker of aging [1,2] and is independently associated with a wide spectrum of negative health-related events (e.g., mobility limitations, falls, institutionalization, and death) [3,4,5,6,7]. Reduced physical performance is also a cardinal element of frailty and sarcopenia [8,9]. Hence, the maintenance of physical performance in old age is recognized as a priority by major public health agencies and a target for interventions during the United Nations Decade of Healthy Ageing [10].

Physical activity (PA) refers to any voluntary muscle contraction that increases energy expenditure above resting values, while physical exercise (PE) involves repetitive, planned, and structured movements to improve or maintain one or more components of physical fitness [11]. The effectiveness of PA and PE for preventing and managing geriatric conditions associated with neuromuscular dysfunction is widely acknowledged [12,13,14,15]. Ramsey et al. [16] examined 112 articles that investigated the associations between PA levels and neuromuscular function in more than 40,000 adults ranging in age from 61 to 88 years. Authors noted that individuals with higher PA levels were stronger and performed better on the five-time sit-to-stand (5STS) test than those with lower levels of PA [16]. Less is known about the association between PA and other physical performance parameters, such as muscle power. This information holds considerable value because, during aging, lower-limb muscle power declines earlier, faster, and to a greater extent than other established physical function metrics (e.g., muscle strength) [17,18]. Moreover, losses in muscle power predict the occurrence of negative health-related events in advanced age [19,20,21].

PE is a large construct that involves a range of exercise modalities that elicit specific changes in muscle mass and function [22]. Strength training, for instance, is commonly performed at moderate-to-high intensities with predominant recruitment of fast-twitch, type II muscle fibers [22]. These fibers generate greater strength and power and are more susceptible to hypertrophy than slow-twitch, type I myofibers. In contrast, endurance activities, such as running and cycling, are usually performed for long periods and involve the activation of type I muscle fibers that are more resistant to fatigue but less qualified to produce tension or change their structure [22]. Nevertheless, studies reported preservation of neuromuscular function also in older adults practicing endurance training [23,24]. Experts in the field have suggested that greater benefits might be achieved by combining PA and PE [11], although no empirical evidence to support this proposition is available.

To fill this gap in knowledge, this study was undertaken to investigate cross-sectional associations of PA and/or PE with commonly used physical performance and anthropometric measures in a large and relatively unselected sample of community-dwelling older men and women.

## 2. Materials and Methods

### 2.1. Study Design and Participants

Data for the present investigation were extracted from the Longevity Check-up 7+ (Lookup 7+) project database. Lookup 7+ is an ongoing, prospective observational study developed by the Department of Geriatrics of the Fondazione Policlinico “Agostino Gemelli” IRCCS at the Università Cattolica del Sacro Cuore (Rome, Italy).

Participants are recruited among those visiting public spaces (e.g., exhibitions, shopping centers) or adhering to prevention campaigns promoted by our institution. Depending on the setting, the project is promoted through various media channels such as newspapers, magazines, and television broadcasting. Visitors are also invited to participate by direct contact. As described previously, to attain a reasonably thorough geographic representation of mainland Italy and its major islands, participant recruitment is carried out in cities of varying sizes: small cities with a population of up to 100,000 persons, medium-sized cities with a population ranging from 100,000 to 250,000 inhabitants, and large cities with a population exceeding 250,000 individuals [25]. In large cities, participants are recruited in various locations to obtain an adequate representation of the sociodemographic characteristics of the population. Exclusion criteria are inability or unwillingness to provide written informed consent, self-reported pregnancy, and inability to perform physical tests.

From 1 June 2015 to 30 October 2021, 13,515 community-dwelling adults 18 years and older were recruited. For this study, analyses were conducted using data from participants 65 and older (*n* = 4119, 53% women). The Lookup 7+ protocol was approved by the Ethics Committee of the Università Cattolica del Sacro Cuore, Rome (Protocol No. A.1220/CE/2011), and each participant provided written informed consent prior to enrolment. The study protocol is detailed elsewhere [26].

The manuscript was prepared according to the STrengthening the Reporting of OBservational studies in Epidemiology (STROBE) guidelines for observational studies [27].

### 2.2. Data Collection

Data relevant to this study were collected by a team of healthcare professionals, including medical doctors, researchers, and dieticians, through standardized questionnaires and objective measurements.

Anthropometric measures encompassed height, weight, and calf circumference. Body height (m) and weight (kg) were measured through a standard stadiometer and an analog medical scale, respectively. The body mass index (BMI) was calculated as the ratio between body weight (kg) and the square of height (m^2^). Calf circumference (cm) was taken on the dominant leg by measuring the largest girth between ankle and knee joints using an anthropometric tape while the participant was in a seated position. Appendicular skeletal muscle mass (ASM) was estimated using the equation proposed by Santos et al. [28]:

$$\begin{array}{cc} \mathrm{ASM}\;\left(\mathrm{kg}\right)= & -10.427+\left(\mathrm{calf}\;\mathrm{circumference}\;\times 0.768\right)-\left(\mathrm{age}\;\times 0.029\right)\\ & \;\;+\left(\mathrm{sex}\;\left[\mathrm{male}=1;\;\mathrm{female}=0\right]\times 7.523\right)\\ & \;\;+\;\mathrm{ethnicity}\;(\mathrm{White}\;=0,\\ & \;\;\mathrm{Black}\;=2.203,\mathrm{Hispanic}\;\mathrm{or}\;\mathrm{Latin}\;=-0.540,\mathrm{other}\;\\ & \;\;=-0.402) \end{array}$$

In addition to unadjusted ASM, the ASM-to-BMI ratio was calculated owing to the stronger association of BMI-adjusted appendicular lean mass (aLM) with negative health-related outcomes in older individuals [29,30]. Indeed, aLM/BMI was indicated by the Foundation for National Institutes of Health (FNIH) sarcopenia project as the preferred criterion for defining clinically relevant low lean mass [31].

Healthy diet was operationalized as a daily consumption of three or more servings (~400 g) of fruit and/or vegetables, which is the minimum amount recommended by the World Health Organization [32]. The daily intake of fruit and vegetables was calculated based on reference tables for the Italian population released by the Italian Society of Nutrition (SINU) [33]. Smoking status was categorized as current smoker (has smoked 100+ cigarettes in their lifetime and currently smokes cigarettes or tobacco surrogate products), never smoked (has never smoked or has smoked <100 cigarettes in their lifetime), or former smoker (has smoked at least 100 cigarettes in their lifetime but had quit at least 28 days before the interview).

Physical performance measures included isometric handgrip strength and the 5STS tests. Both tests were administered by trained assessors according to standardized protocols. Handgrip strength was measured using a hydraulic handheld dynamometer (North Coast Medical, Inc., Morgan Hill, CA, USA) while the participant was seated on a chair with the shoulder and the wrist in a neutral position and the elbow near the trunk and flexed. One familiarization trial was allowed before the actual test was conducted. Handgrip strength was measured in both hands, and the higher value (kg) was recorded and used for the analysis. For the 5STS test, participants were instructed to stand up completely from an armless chair five consecutive times as fast as possible, with their arms crossed over their chest. A stopwatch was used to measure the time (s) needed to complete the task.

Absolute, relative (adjusted by body weight), allometric (adjusted by height squared), and specific (adjusted by ASM) muscle power values of the lower extremities were calculated according to the equation proposed by Alcazar et al. [34]:

$$\begin{array}{c} \mathrm{Absolute}\;\mathrm{muscle}\;\mathrm{power}\;\left(W\right)\\ \;\;\;\;\;\;\;=\frac{\mathrm{Body}\;\mathrm{weight}\;\;\left(\mathrm{kg}\right)\;\times \;g\;\times \;\left[\mathrm{height}\;\left(m\right)\;\times \;0.5\;-\;\mathrm{chair}\;\mathrm{height}\;\left(m\right)\right]}{\left(\left[\frac{\left(5\mathrm{STS}\;\mathrm{test}\;\mathrm{time}\;\left(s\right)\right)}{\mathrm{no}.\;\mathrm{of}\;\mathrm{STS}\;\mathrm{repetitions}}\right]\;\times \;0.5\right)} \end{array}$$

where *g* is the acceleration due to gravity (i.e., 9.80352 m·s^−2^)

$$\mathrm{Relative}\;\mathrm{muscle}\;\mathrm{power}\;\left(W/\mathrm{kg})\right)=\frac{\mathrm{Absolute}\;\mathrm{muscle}\;\mathrm{power}\;\left(W\right)}{\mathrm{Body}\;\mathrm{weight}\;\left(\mathrm{kg}\right)}$$

$$\mathrm{Allometric}\;\mathrm{muscle}\;\mathrm{power}\;\left({W/m}^{2}\right)=\frac{\mathrm{Absolute}\;\mathrm{muscle}\;\mathrm{power}\;\left(W\right)}{\mathrm{Height}\;\left(m^{2}\right)}$$

$$\mathrm{Specific}\;\mathrm{muscle}\;\mathrm{power}\;\left(W/\mathrm{cm}\right)=\frac{\mathrm{Absolute}\;\mathrm{muscle}\;\mathrm{power}\;\left(W\right)}{\;\mathrm{ASM}\;\left(\mathrm{kg}\right)}$$

Regular participation in PA or PE was operationalized as engagement in activities for a minimum of 30 min at least twice weekly during the past year. To collect this information, participants answered the question: “Did you perform at least 30 min of physical activity or exercise twice a week or more often during the last 12 months? If so, please specify the activity”. The following activities were considered: (a) light walking (for PA), (b) running, cycling, or swimming, and (c) strength training with or without stretching exercises. Accordingly, participants were categorized into (a) inactive (did not perform at least 30 min of PA or PE at least twice weekly); (b) light walkers; (c) practicing running/cycling/swimming; (d) practicing strength training +/− stretching; and (e) practicing light walking + any PE. The rationale for this categorization lies in the fact that PA (e.g., walking) and PE, although often considered synonyms, are two distinct activity modalities that differ according to their level of organization [11]. The PE domain was further categorized into endurance or “aerobic-based” activities that are usually performed at low-to-moderate intensity and high volume (e.g., running, swimming, cycling) and activities performed at moderate-to-high intensities and low volume (strength training) [22]. As mentioned earlier, endurance and strength training involve the recruitment of distinct muscle fibers and elicit specific neuromuscular, cardiovascular, and metabolic adaptations [22]. Stretching exercises were included because some participants specified that they performed strength training and flexibility exercises.

The interview did not include questions related to actual exercise frequency, volume, or intensity.

### 2.3. Statistical Analysis

Continuous variables are expressed as mean ± standard deviation (SD), while categorical variables are shown as absolute numbers (%). According to the Kolmogorov–Smirnov test, data were not normally distributed. Non-Gaussian distribution may be ignored if large sample sizes (>30–40 participants) with values representative of a “real population” are analyzed [35,36]. Participant characteristics were first described according to sex and then according to activity categories in men and women separately. Five activity groups were considered: physically inactive, light walking, running/cycling/swimming, strength training with or without stretching exercises, and light walking plus any kind of exercise. Differences in proportions between groups were analyzed by chi-squared (χ²) statistics. Student’s *t*-tests or analysis of variance (ANOVA) with Bonferroni’s post hoc tests, when appropriate, were used to analyze differences in means. All tests were two-sided, with statistical significance set at *p* < 0.05. Logistic binary regression analysis was used to explore the association of PA and/or PE with measures of physical performance and anthropometry. Separate models were built for male and female participants. Handgrip strength, 5STS, and ASM were dichotomized according to cutoff values proposed by the European Working Group on Sarcopenia 2 (EWGSOP2) [37], while median values were used for lower-limb muscle power measures. Results are reported as odds ratios with 95% confidence intervals. To be considered as an independent predictor, variables were required to have a *p* < 0.05. Models were adjusted for age, smoking status, and healthy diet in a single step (enter method). Model fit was evaluated using the Hosmer–Lemeshow test. All variables had low to moderate correlations (0.2 to 0.6), except for 5STS and relative muscle power, suggesting little multicollinearity. All analyses were performed using the SPSS software version 23.0 (SPSS Inc., Chicago, IL, USA).

## 3. Results

### 3.1. Study Population

The main characteristics of the 4119 participants according to sex are listed in Table 1. Men were slightly older, more frequently physically active, had greater BMI values, and were more often on a healthy diet than women. Among physically active participants, the proportions of men practicing light walking or engaged in running/cycling/swimming exercises were greater than in women. Compared with female participants, men had greater absolute and BMI-adjusted ASM and handgrip strength, performed better on the 5STS, and had greater lower-limb muscle power.

Participant characteristics according to activity categories are shown in Table 2 and Table 3 in men and women, respectively. Compared with inactive participants, physically active men and women were generally younger and non-smokers, had lower BMI values, were more frequently on a healthy diet, had greater handgrip strength and muscle power, and performed better on the 5STS test. Physically active men had greater BMI-adjusted ASM than their physically inactive counterparts (Table 2). Among women, only those practicing light walking plus PE had greater absolute ASM than physically inactive peers (Table 3).

### 3.2. Associations between Physical Activity Habits and Measures of Physical Performance and Anthropometry

Results of logistic binary regression in men and women are shown in Table 4 and Table 5, respectively. Additional results are reported in Table S1 for men and Table S2 for women. After adjustment for potential confounders (i.e., age, smoking status, and healthy diet), compared with physically inactive men, those engaged in running, swimming, or cycling were more likely to have greater handgrip strength. Regardless of activity type, physically active men had greater probabilities of performing better on the 5STS test and having greater relative muscle power. Men practicing PE, except for those engaged in strength training, had higher odds for greater BMI-adjusted handgrip strength and ASM. Only those practicing running, swimming, or cycling were more likely to have greater absolute and allometric muscle power. Physically active men, except for light walkers, had a greater likelihood of greater specific muscle power. When compared with light walkers, men engaged in running, swimming, or cycling were more likely to have greater absolute, allometric, and specific muscle power. Those in the walking + PE group had more chances to have greater BMI-adjusted handgrip strength and ASM, better performance on the 5STS test, and greater relative and specific muscle power.

Among female participants, engagement in walking activity plus any PE was associated with a higher likelihood of greater handgrip strength compared with physically inactive peers. Physically active women, except for those engaged in strength training, had greater probabilities of better performance on the 5STS test. Only PE groups were more likely to have greater absolute muscle power. Light walking and all PE groups had a higher likelihood of greater BMI-adjusted handgrip strength and relative muscle power. Engagement in running/cycling/swimming exercises or walking plus PE was associated with greater allometric and specific muscle power. Women practicing light walking with or without PE had higher chances of greater BMI-adjusted ASM. Compared with the light walking group, all PE groups had greater probabilities of better handgrip strength, 5STS performance, absolute, relative, and allometric muscle power, and greater ASM, either absolute or BMI-adjusted. Only women practicing running, swimming, or cycling had a higher likelihood of greater specific muscle power.

Engagement in light walking plus PE was not significantly associated with better physical performance or greater ASM in comparison to PE alone in either men or women.

## 4. Discussion

The main findings of this study indicate that engagement in regular walking activity and/or PE is significantly associated with better physical performance and greater ASM in older adults living in the community. Associations were influenced by the type of activity, physical performance assessment tool, anthropometric parameter, and sex. Male and female participants practicing any type of activity showed better physical performance and greater ASM than their physically inactive peers. Compared with light walkers, only men engaged in both walking activity and PE had better 5STS performance, greater BMI-adjusted handgrip strength and ASM, and greater relative and specific muscle power. In women, the combination of light walking and PE was only associated with greater handgrip strength compared with physically inactive peers. Female participants engaged in running/cycling/swimming exercises or in light walking plus any type of PE had better physical performance and ASM than those only practicing light walking.

Our results are supported by previous investigations. In the Lifestyle Interventions and Independence for Elders Pilot (LIFE-P) study, a 12-month structured PA program improved physical performance, as assessed through the short physical performance battery (SPPB) and 400 m walking speed, in community-dwelling older adults at risk of mobility disability [38]. These findings were expanded by Bernabei et al. [15], who reported that a multicomponent intervention including structured PA with technological support and nutritional counseling attenuated the loss of handgrip strength and aLM, either absolute or adjusted by BMI, in older women with physical frailty and sarcopenia over up to 36 months of follow-up. The intervention also reduced the incidence of inability to walk 400 m in both sexes [15]. In a study involving more than 400 older adults, Gonçalves et al. [39] showed that a 6-month multicomponent exercise program encompassing strength training and balance exercises enhanced mobility and balance. More recently, Coelho-Junior and Uchida [40] found that a 4-month resistance training program ameliorated muscle strength and power in community-dwelling older adults. Systematic reviews and meta-analyses have reported similar findings [41,42]. 

In our study, men and women who practiced PE were stronger and had greater lower-limb muscle power than those who only walked. A possible explanation for this finding is that PE involves the performance of body movements that are organized, planned, and structured according to the variables of exercise training (e.g., intensity, volume, rest) [11]. Strength training [41,42] and running/swimming/cycling [43,44,45] may improve neuromuscular function, including muscle strength and power, through multi-joint exercises performed at adequate intensity and velocity. Conversely, light walking is performed at low intensity, which only recruits type I muscle fibers that are more resistant to fatigue but have a lower capacity to generate tension and power [46]. 

We also observed that engagement in light walking plus PE was associated with better functional and anthropometric parameters than walking activity alone. Sex-specific associations were detected. Increasing activity levels enhances physical performance in many ways. PA is significantly associated with bone mineral density [47], brain function [48], and metabolic health [49] and is negatively correlated with inflammation [50,51], arterial stiffness [50], fatigue [51], and the presence of multimorbidity [52]. All these variables are directly or indirectly associated with physical performance [53].

Another interesting finding of our study is that light walking plus PE was associated with BMI-adjusted ASM in men only. Similar results were found by Shibata et al. [54] in community-dwelling Japanese older adults. The practice of PA might amplify the effects of PE on muscle mass by reducing the accumulation of adipose tissue. Excess adiposity is associated with a muscular catabolic environment through metabolic and inflammatory signaling [55]. Differences between sexes might be explained by the amount and intensity of PA [56]. Women traditionally spend more time in domestic work than men, who instead are more frequently involved in occupational and recreational tasks [57]. Other factors possibly associated with energy metabolism, such as diet quality and sleep patterns, were not controlled for in this study. 

This study is not free of limitations. First, PE variables were not controlled for in the analysis. This aspect is important because exercise frequency, volume, and intensity impact physical performance and muscle mass. Second, muscle power and ASM were estimated through equations rather than being measured directly. Third, our sample was composed of relatively young community-dwelling Caucasian older adults, and extrapolation to individuals in other conditions should be made with caution. Fourth, participants were evaluated while they were attending an event. Thus, the possibility that the evaluation setting could have influenced physical performance results cannot be ruled out. Fifth, information on chronic diseases (e.g., osteoarthritis) or medications (e.g., corticosteroids) that could impact musculoskeletal health was not available. The collection of a detailed medical history would substantially increase the duration of the assessments, making them unsuitable for the unconventional settings where the research is conducted. Sixth, the use of structured instruments to assess the main variables might provide different results. Finally, the cross-sectional design of this study does not allow any inference to be drawn on the time course of changes in the variables considered and on cause–effect relationships.

## 5. Conclusions

The results of this study indicate that regular PA and/or PE are significantly associated with functional and anthropometric parameters in community-dwelling older adults. Specific patterns of associations were detected depending on the type of activity, physical function assessment tool, anthropometric parameter, and participant sex. Our findings suggest that walking activity is already associated with better physical performance and greater ASM than physical inactivity. Stronger associations were observed in participants practicing PE. Those who engaged in light walking plus any kind of PE had better physical function and greater ASM than light walkers. 

## Figures and Tables

**Table 1:** Main characteristics of study participants according to sex.

	Men (*n* = 1897)	Women (*n* = 2222)	*p*
Age, years	72.8 ± 5.8	72.1 ± 5.4	<0.001
BMI, kg/m²	26.8 ± 3.5	26.1 ± 4.4	<0.001
Healthy diet, *n* (%)	450 (23.7)	446 (20.1)	0.006
Current smokers, *n* (%)	253 (13.3)	256 (11.5)	0.100
Physically inactive, *n* (%)	1001 (52.8)	1390 (62.6)	<0.001
Light walking, *n* (%)	506 (26.7)	515 (23.2)	0.011
Running/cycling/swimming, *n* (%)	219 (11.5)	171 (7.7)	<0.001
Strength training +/− stretching, *n* (%)	32 (1.7)	46 (2.1)	0.430
Light walking + any type of exercise, *n* (%)	139 (7.3)	100 (4.5)	<0.001
Handgrip strength, kg	35.2 ± 7.9	20.5 ± 5.4	<0.001
Handgrip strength/BMI	1.3 ± 0.34	0.80 ± 0.24	<0.001
5STS, s	8.8 ± 2.5	9.4 ± 2.9	<0.001
Muscle power			
Absolute, W	326.4 ± 95.7	220.6 ± 69.0	<0.001
Relative, W/kg	4.17 ± 1.07	3.43 ± 0.95	<0.001
Allometric, W/m^2^	111.6 ± 30.9	88.5 ± 25.5	<0.001
Specific, W/kg	14.7 ± 4.1	16.7 ± 5.8	<0.001
Calf circumference, cm	35.8 ± 3.2	34.1 ± 3.3	<0.001
Appendicular skeletal muscle mass, kg	22.4 ± 2.5	13.8 ± 2.6	<0.001
Appendicular skeletal muscle mass/BMI	0.84 ± 0.10	0.53 ± 0.09	<0.001

Data are shown as absolute numbers (percentages) for categorical variables and mean ± standard deviation for continuous variables. Abbreviations: 5STS, five-time sit-to-stand; BMI, body mass index.

**Table 2:** Main characteristics of male participants according to activity categories (*n* = 1897).

	Inactive (*n* = 1001)	Walking (*n* = 506)	Running/Cycling/Swimming (*n* = 219)	Strength Training +/− Stretching (*n* = 32)	Walking + Any Exercise (*n* = 139)
Age, years	73.0 ± 6.0	73.1 ± 5.5	71.6 ± 5.9 ^ab^	72.5 ± 6.4	71.7 ± 5.6
BMI, kg/m²	27.4 ± 3.7	26.3 ± 3.3 ^a^	26.1 ± 3.1 ^a^	27.2 ± 4.6	25.6 ± 3.2 ^a^
Healthy diet, *n* (%)	264 (26.4)	96 (19.0)	51 (23.3)	3 (9.4)	36 (25.9)
Current smokers, *n* (%)	138 (13.8)	64 (12.6)	31 (14.2)	5 (15.6)	15 (10.8)
Handgrip strength, kg	34.5 ± 8.1	35.4 ± 7.5	36.6 ± 7.2 ^ab^	35.6 ± 8.4	36.6 ± 8.3 ^a^
Handgrip strength/BMI	1.27 ± 0.32	1.35 ± 0.31 ^a^	1.41 ± 0.32 ^a^	1.32 ± 0.32	1.45 ± 0.48 ^ab^
5STS, s	9.20 (2.80)	8.78 (2.15) ^a^	8.15 (2.0) ^ab^	7.65 (1.45) ^a^	8.09 (2.02) ^ab^
Muscle power					
Absolute, W	318.5 ± 94.9	322.5 ± 93.0	354.2 ± 95.2 ^ab^	359.7 ± 90.9	330.8 ± 98.5
Relative, W/kg	3.99 ± 1.01	4.24 ± 1.07 ^ab^	4.56 ± 1.11 ^ab^	4.64 ± 1.02	4.47 ± 1.19
Allometric, W/m^2^	109.3 ± 30.5	110.8 ± 31.4	119.3 ± 31.0 ^ab^	124.6 ± 30.5	112.7 ± 29.3
Specific, W/kg	14.2 ± 4.0	14.5 ± 4.0	15.9 ± 4.2 ^ab^	16.7 ± 4.6	15.0 ± 4.2
Calf circumference, cm	35.8 ± 3.3	35.8 ± 3.1	35.6 ± 2.8	35.5 ± 3.7	35.4 ± 3.3
Appendicular skeletal muscle mass, kg	22.5 ± 2.5	22.4 ± 2.4	22.3 ± 2.2	22.2 ± 2.9	22.2 ± 2.6
Appendicular skeletal muscle mass/BMI	0.82 ± 0.09	0.85 ± 0.09 ^a^	0.86 ± 0.10 ^a^	0.82 ± 0.09 ^a^	0.87 ± 0.14 ^a^

Data are shown as absolute numbers (percentages) for categorical variables and mean ± standard deviation for continuous variables. ^a^ *p* < 0.05 vs. inactive; ^b^ *p* < 0.05 vs. walking. Abbreviations: 5STS, five-time sit-to-stand, BMI, body mass index.

**Table 3:** Main characteristics of female participants according to activity categories (*n* = 2222).

	Inactive (*n* = 1390)	Walking (*n* = 515)	Running/Cycling/Swimming (*n* = 171)	Strength Training +/− Stretching (*n* = 46)	Walking + Any Exercise (*n* = 100)
Age, years	72.5 ± 5.6	71.6 ± 5.1 ^a^	71.4 ± 4.8	72.2 ± 5.6	70.2 ± 4.6 ^a^
BMI, kg/m²	26.6 ± 4.7	25.2 ± 3.6 ^a^	25.7 ± 4.4	25.6 ± 4.0	24.5 ± 3.2 ^a^
Healthy diet, *n* (%)	327 (23.5) *	75 (14.6)	26 (15.2)	5 (10.9)	13 (13.0)
Current smokers, *n* (%)	163 (11.7)	51 (9.9)	23 (13.5)	3 (6.5)	16 (16.0)
Handgrip strength, kg	20.2 ± 5.4	20.7 ± 5.1	21.0 ± 5.2	21.0 ± 6.5	22.1 ± 5.8
Handgrip strength/BMI	0.77 ± 0.23	0.83 ± 0.23 ^a^	0.83 ± 0.24 ^a^	0.83 ± 0.25	0.91 ± 0.27 ^ab^
5STS, s	9.9 (3.2)	9.0 (2.2) ^a^	8.4 (2.4) ^a^	7.9 (1.7) ^a^	7.8 (1.7) ^ab^
Muscle power					
Absolute, W	214.7 ± 67.2	224.0 ± 64.9	242.0 ± 70.8 ^ab^	251.0 ± 97.6	246.8 ± 73.7 ^a^
Relative, W/kg	3.28 ± 0.93	3.59 ± 0.85 ^ab^	3.87 ± 1.04 ^ab^	3.90 ± 1.05	4.04 ± 1.02 ^ab^
Allometric, W/m^2^	86.4 ± 25.3	89.4 ± 23.4	97.4 ± 27.0 ^ab^	96.2 ± 27.4	98.5 ± 23.8 ^a^
Specific, W/kg	16.2 ± 5.5	17.1 ± 5.8	18.7 ± 7.8 ^a^	18.6 ± 5.6	18.4 ± 5.5
Calf circumference, cm	34.1 ± 3.5	34.0 ± 3.0	33.9 ± 2.9	34.2 ± 2.70	34.0 ± 2.8
Appendicular skeletal muscle mass, kg	13.8 ± 2.7	13.8 ± 2.3 ^a^	13.7 ± 2.3	13.9 ± 2.1	13.8 ± 2.2 ^a^
Appendicular skeletal muscle mass/BMI	0.52 ± 0.08	0.55 ± 0.09	0.54 ± 0.09	0.55 ± 0.08	0.56 ± 0.07

Data are shown as absolute numbers (percentages) for categorical variables and mean ± standard deviation for continuous variables. ^a^ *p* < 0.05 vs. inactive; ^b^ *p* <0.05 vs. walking; * *p* < 0.05 at χ^2^ test. Abbreviations: 5STS, five-time sit-to-stand; BMI, body mass index.

**Table 4:** Associations of physical activity habits with physical performance measures and estimated appendicular skeletal muscle mass in male participants (*n* = 1897).

	Univariate β (95% CI)	Adjusted (95% CI)		Univariate β (95% CI)	Adjusted (95% CI)		Univariate β (95% CI)	Adjusted (95% CI)		Univariate β (95% CI)	Adjusted (95% CI)
Handgrip strength											
Physically inactive	1.00 (Reference)	1.00 (Reference)	Walking	1.00 (Reference)	1.0 (Reference)	Running/cycling/swimming	1.00 (Reference)	1.00 (Reference)	Strength +/− stretching	1.00 (Reference)	1.00 (Reference)
Walking	0.22 (−0.75, 1.34)	0.58 (−0.36, 1.52)	Running/cycling/swimming	2.00 (0.73, 3.27)	0.93 (−0.26, 2.13)	Walking + exercise	0.00 (−1.78, 1.78)	−0.03 (−1.17, 1.65)	Walking + exercise	1.37 (−3.31, 6.04)	0.76 (−3.61, 5.12)
Running/cycling/swimming	2.29 (1.06, 3.52)	1.37 (0.24, 2.50)	Strength +/− stretching	0.63 (−3.12, 4.38)	−0.32 (−3.74, 3.08)						
Strength +/− stretching	0.925 (−3.33, 5.18)	0.71 (−3.13, 4.55)	Walking + exercise	2.00 (0.38, 3.62)	1.31 (−0.17, 2.78)						
Walking + exercise	2.29 (0.66, 3.93)	1.31 (−0.17, 2.78)									
5STS											
Physically inactive	1.00 (Reference)	1.00 (Reference)	Walking	1.00 (Reference)	1.00 (Reference)	Running/cycling/swimming	1.00 (Reference)	1.00 (Reference)	Strength +/− stretching	1.00 (Reference)	1.00 (Reference)
Walking	9.31 (9.13, 9.50)	−0.65 (−0.98, −0.30)	Running/cycling/swimming	−0.54 (−0.93, −0.16)	−0.36 (−0.74, 0.03)	Walking + exercise	−0.08 (−0.56, 0.39)	−0.06 (−0.52, 0.41)	Walking + exercise	0.42 (−0.71, 1.56)	0.46 (−0.66, 1.59)
Running/cycling/swimming	−1.12 (−1.54, −0.70)	−0.938 (−1.34, −0.53)	Strength +/− stretching	−1.05 (−2.26, 0.17)	−0.91 (−2.10, 0.29)						
Strength +/− stretching	−1.63 (−3.14, −0.11)	−1.54 (−2.99, −0.08)	Walking + exercise	−0.63 (−1.11, −0.14)	−0.93 (−1.53, −0.46)						
Walking + exercise	−1.20 (−1.76, −0.65)	−0.99 (−1.53, −0.46)									
Absolute muscle power											
Physically inactive	1.00 (Reference)	1.00 (Reference)	Walking	1.00 (Reference)	1.00 (Reference)	Running/cycling/swimming	1.00 (Reference)	1.0 (Reference)	Strength +/− stretching	1.00 (Reference)	1.00 (Reference)
Walking	4.08 (−8.433, 16.599)	7.77 (−4.00, 19.54)	Running/cycling/swimming	31.65 (14.93, 48.36)	22.46 (5.90, 39.02)	Walking + exercise	−10.93 (−33.55, 11.69)	−11.01 (−33.32, 11.30)	Walking + exercise	−16.41 (−71.79, 38.97)	−15.83 (−69.68, 38.01)
Running/cycling/swimming	35.73 (21.15, 50.31)	27.97 (14.09, 41.85)	Strength +/− stretching	37.12 (−12.90, 87.14)	27.29 (−20.68, 75.26)						
Strength +/− stretching	41.21 (−8.98, 91.40)	37.64 (−9.15, 84.44)	Walking + exercise	20.71 (−0.18, 41.61)	16.11 (−1.90, 34.13)						
Walking + exercise	24.80 (5.60, 43.99)	16.11 (−1.90, 34.13)									
ASM											
Physically inactive	1.00 (Reference)	1.00 (Reference)	Walking	1.00 (Reference)	1.00 (Reference)	Running/cycling/swimming	1.00 (Reference)	1.00 (Reference)	Strength +/− stretching	1.00 (Reference)	1.00 (Reference)
Walking	−0.15 (−0.48, 0.19)	−0.11 (−0.44, 0.27)	Running/cycling/swimming	0.11 (−0.32, 0.54)	−0.16 (−0.57, 0.26)	Walking + exercise	−0.25 (−0.80, 0.30)	−0.24 (−0.76, 0.28)	Walking + exercise	0.34 (−1.16, 1.83)	0.14 (−1.27, 1.55)
Running/cycling/swimming	−0.04 (−0.42, 0.35)	−0.20 (−0.58, 0.18)	Strength +/− stretching	−0.48 (−1.83, 0.86)	−0.69 (−1.95, 0.65)						
Strength +/− stretching	−0.63 (−1.97, 0.71)	−0.64 (−1.96, 0.69)	Walking + exercise	−0.14 (−0.70, 0.41)	−0.45 (−0.95, 0.06)						
Walking + exercise	−0.29 (−0.80, 0.22)	−0.45 (−0.95, 0.06)									

Models were adjusted for age, smoking habits, and healthy diet. Gray-shadowed cells denote statistical significance. Abbreviations: 5STS, five-time sit-to-stand; ASM, appendicular skeletal muscle mass; CI, confidence interval.

**Table 5:** Associations of physical activity habits with physical performance measures and estimated appendicular skeletal muscle mass in female participants (*n* = 2222).

	Univariate β (95% CI)	Adjusted (95% CI)		Univariate β (95% CI)	Adjusted (95% CI)		Univariate β (95% CI)	Adjusted (95% CI)		Univariate β (95% CI)	Adjusted (95% CI)
Handgrip strength											
Physically inactive	1.00 (Reference)	1.00 (Reference)	Walking	1.00 (Reference)	1.00 (Reference)	Running/cycling/swimming	1.00 (Reference)	1.00 (Reference)	Strength +/− stretching	1.00 (Reference)	1.00 (Reference)
Walking	0.25 (−0.39, 0.89)	−0.00 (−0.62, 0.61)	Running/cycling/swimming	0.41 (−0.50, 1.35)	2.49 (1.23, 3.70)	Walking + exercise	1.26 (−0.53, 3.05)	1.11 (−0.66, 2.88)	Walking + exercise	2.23 (−1.76, 6.22)	1.60 (−2.35, 5.54)
Running/cycling/swimming	0.66 (−0.25, 1.57)	0.22 (−0.65, 1.009)	Strength +/− stretching	2.27 (−0.41, 4.95)	1.60 (−1.82, 5.01)						
Strength +/− stretching	2.52 (−0.27, 5.31)	2.48 (−0.19, 5.15)	Walking + exercise	2.51 (1.20, 3.82)	3.70 (2.15, 5.25)						
Walking + exercise	2.76 (1.48, 4.05)	2.04 (0.79, 3.28)									
5STS											
Physically inactive	1.00 (Reference)	1.00 (Reference)	Walking	1.00 (Reference)	1.00 (Reference)	Running/cycling/swimming	1.00 (Reference)	1.00 (Reference)	Strength +/− stretching	1.00 (Reference)	1.00 (Reference)
Walking	−1.00 (−1.41, −0.65)	−0.86 (−1.22, −0.49)	Running/cycling/swimming	−0.59 (−1.01, −0.13)	−0.45 (−0.74, −0.16)	Walking + exercise	−0.24 (−0.62, 0.14)	−0.20 (−0.57, 0.17)	Walking + exercise	−0.01 (−0.75, 0.73)	0.04 (−0.69, 0.78)
Running/cycling/swimming	−1.621 (−2.186, −1.0056)	−1.320 (−1.857, −0.783)	Strength +/− stretching	−0.614 (−1.778, 0.551)	−0.776 (−1.596, 0.043)						
Strength +/− stretching	−1.65 (−3.29, 0.00)	−1.54 (−3.10, 0.03)	Walking + exercise	−1.005 (−1.67, −0.49)	−0.67 (−1.00, −0.31)						
Walking + exercise	−2.09 (−2.86, −1.31)	−1.66 (−2.40, −0.91)									
Absolute muscle power											
Physically inactive	1.00 (Reference)	1.00 (Reference)	Walking	1.00 (Reference)	1.00 (Reference)	Running/cycling/swimming	1.00 (Reference)	1.00 (Reference)	Strength +/− stretching	1.00 (Reference)	1.00 (Reference)
Walking	9.27 (1.20, 17.34)	5.41 (−2.28, 13.09)	Running/cycling/swimming	17.99 (4.97, 31.00)	33.83 (21.25, 46.41)	Walking + exercise	−2.11 (−20.41, 16.19)	−3.13 (−21.28, 15.02)	Walking + exercise	3.702 (−36.19, 43.60)	0.31 (−39.38, 40.00)
Running/cycling/swimming	27.26 (15.56, 38.95)	20.48 (9.45, 31.48)	Strength +/− stretching	27.01 (−6.55, 60.56)	29.92 (−4.32, 64.15)						
Strength +/− stretching	36.28 (2.82, 69.73)	33.40 (2.08, 64.73)	Walking + exercise	26.19 (9.20, 43.18)	31.18 (15.45, 46.90)						
Walking + exercise	35.46 (19.48, 51.44)	25.289 (10.17, 40.40)									
ASM											
Physically inactive	1.00 (Reference)	1.00 (Reference)	Walking	1.00 (Reference)	1.00 (Reference)	Running/cycling/swimming	1.00 (Reference)	1.00 (Reference)	Strength +/− stretching	1.00 (Reference)	1.00 (Reference)
Walking	−0.17 (−0.49, 0.15)	−0.25 (−0.56, 0.07)	Running/cycling/swimming	0.01 (−0.44, 0.46)	1.001 (0.36, 1.66)	Walking + exercise	0.20 (−0.67, 1.007)	0.20 (−0.62, 1.006)	Walking + exercise	1.33 (−0.55, 3.19)	1.13 (−0.70, 2.95)
Running/cycling/swimming	−0.16 (−0.62, 0.29)	−0.30 (−0.74, 0.15)	Strength +/− stretching	−0.04 (−1.26, 1.19)	−0.13 (−2.00, 1.69)						
Strength +/− stretching	0.21 (−1.57, 1.16)	−0.25 (−1.59, 1.008)	Walking + exercise	0.20 (−0.41, 0.80)	1.28 (0.46, 2.10)						
Walking + exercise	0.03 (−0.62, 0.67)	−0.15 (−0.79, 0.49)									

Models were adjusted for age, smoking habits, and healthy diet. Gray-shadowed cells denote statistical significance. Abbreviations: 5STS, five-time sit-to-stand; ASM, appendicular skeletal muscle mass; CI, confidence interval.

## Data Availability

Data are available upon reasonable request.

## Supplementary Materials

The following supporting information can be downloaded at https://www.mdpi.com/article/10.3390/jcm12247521/s1, Table S1: Associations of physical activity habits with physical performance measures and estimated appendicular skeletal muscle mass in male participants (*n* = 1897); Table S2: Associations of physical activity habits with physical performance measures and estimated appendicular skeletal muscle mass in female participants (*n* = 2222).
