# Supplementary material for: Association of Physical Activity and Exercise with Physical Performance and Muscle Mass in Older Adults: Results from the Longevity Check-Up (Lookup) 7+ Project
Source: J Clin Med. 2023 Dec 5;12(24):7521. doi: 10.3390/jcm12247521 (PMC10744185; doi:10.3390/jcm12247521)
Supplement: Supplementary file 1 [file jcm-12-07521-s001.zip › jcm-2709817-supplementary.pdf]

Table S1. Associations of physical activity habits with physical performance measures and estimated appendicular skeletal muscle mass in male participants (n = 1897).

	**Univariate β (95% CI)**	**Adjusted (95% CI)**		**Univariate β (95% CI)**	**Adjusted (95% CI)**		**Univariate β (95% CI)**	**Adjusted (95% CI)**		**Univariate β (95% CI)**	**Adjusted (95% CI)**
Handgrip strength/BMI											
Physically inactive	1.00 (Reference)	1.00 (Reference)	Walking	1.00 (Reference)	1.00 (Reference)	Running/cycling/swimming	1.00 (Reference)	1.00 (Reference)	Strength +/– stretching	1.00 (Reference)	1.00 (Reference)
Walking	0.07 (0.03, 0.12)	0.09 (0.04, 0.13)	Running/cycling/swimming	0.09 (0.03, 0.15)	0.05 (−0.00, 0.11)	Walking + exercise	0.02 (−0.07, 0.12)	0.01 (−0.08, 0.11)	Walking + exercise	0.11 (−0.18, 0.40)	0.05 (−0.24, 0.33)
Running/cycling/swimming	0.16 (0.11, 0.22)	0.14 (0.09, 0.18)	Strength +/– stretching	0.01 (−0.17, 0.18)	−0.02 (−0.18, 0.14)						
Strength +/– stretching	0.08 (−0.10, 0.26)	0.068 (−0.10, 0.24)	Walking + exercise	0.11 (0.03, 0.20)	0.16 (0.09, 0.23)						
Walking + exercise	0.19 (0.11, 0.26)	0.16 (0.09, 0.23)									
Relative muscle power											
Physically inactive	1.00 (Reference)	1.00 (Reference)	Walking	1.00 (Reference)	1.00 (Reference)	Running/cycling/swimming	1.00 (Reference)	1.00 (Reference)	Strength +/– stretching	1.00 (Reference)	1.00 (Reference)
Walking	0.25 (0.11, 0.38)	0.28 (0.15, 0.41)	Running/cycling/swimming	0.32 (0.19, 0.52)	0.23 (0.04, 0.43)	Walking + exercise	0.03 (−0.24, 0.29)	0.02 (−0.24, 0.29)	Walking + exercise	−0.08 (−0.73, 0.57)	−0.07 (−0.70, 0.57)
Running/cycling/swimming	0.57 (0.41, 0.73)	0.50 (0.34, 0.65)	Strength +/– stretching	0.43 (−0.15, 1.00)	0.35 (−0.22, 0.91)						
Strength +/– stretching	0.66 (0.14, 1.21)	0.64 (0.13, 1.15)	Walking + exercise	0.38 (0.11, 0.59)	0.51 (0.31, 0.71)						
Walking + exercise	0.594 (0.387, 0.802)	0.512 (0.313, 0.710)									
Allometric muscle power											
Physically inactive	1.00 (Reference)	1.00 (Reference)	Walking	1.00 (Reference)	1.00 (Reference)	Running/cycling/swimming	1.00 (Reference)	1.00 (Reference)	Strength +/– stretching	1.00 (Reference)	1.00 (Reference)
Walking	1.54 (−2.53, 5.63)	2.63 (−1.25, 6.51)	Running/cycling/swimming	8.47 (2.91, 14.04)	5.63 (0.09, 11.13)	Walking + exercise	−2.43 (−9.63, 4.77)	−2.53 (−9.66, 4.60)	Walking + exercise	−7.77 (−24.80, 9.26)	−7.12 (−23.76, 9.52)
Running/cycling/swimming	10.03 (5.33, 14.73)	7.74 (3.22, 12.27)	Strength +/– stretching	13.81 (−3.08, 30.70)	10.84 (−5.51, 27.18)						
Strength +/– stretching	15.37 (−0.78, 31.51)	14.41 (−0.85, 29.68)	Walking + exercise	6.04 (−0.82, 12.91)	5.04 (−0.78, 10.86)						
Walking + exercise	7.60 (1.47, 13.73)	5.04 (−0.78, 10.86)									

Specific muscle power											
Physically inactive	1.00 (Reference)	1.00 (Reference)	Walking	1.00 (Reference)	1.00 (Reference)	Running/cycling/swimming	1.00 (Reference)	1.00 (Reference)	Strength +/– stretching	1.00 (Reference)	1.00 (Reference)
Walking	0.28 (–0.25, 0.80)	0.39 (–0.11, 0.90)	Running/cycling/swimming	1.38 (0.65, 2.10)	1.16 (0.42, 1.89)	Walking + exercise	–0.24 (–1.25, 0.77)	–0.25 (–1.26, 0.76)	Walking + exercise	–1.11 (–3.61, 1.38)	–0.90 (–3.38, 1.57)
Running/cycling/swimming	1.65 (1.00, 2.27)	1.39 (0.79, 1.99)	Strength +/– stretching	2.25 (0.11, 4.40)	1.95 (–0,17, 4.06)						
Strength +/– stretching	2.53 (0.41, 4,64)	2.37 (0.36, 4.37)	Walking + exercise	1.14 (0.24, 2.04)	1.11 (0.33, 1.89)						
Walking + exercise	1.41 (0.60, 2.23)	1.11 (0.33, 1.19)									
Calf circumference											
Physically inactive	1.00 (Reference)	1.00 (Reference)	Walking	1.00 (Reference)	1.00 (Reference)	Running/cycling/swimming	1.00 (Reference)	1.00 (Reference)	Strength +/– stretching	1.00 (Reference)	1.00 (Reference)
Walking	–0.18 (–0.61, 0.26)	–0.1 (–0.57, 0.29)	Running/cycling/swimming	0.06 (–0.48, 0.61)	–0.20 (–0.74, 0.34)	Walking + exercise	–0.33 (–1.00, 0.38)	–0.13 (-0.99, 0.36)	Walking + exercise	0.41 (–1.50, 2.32)	0.18 (–1.66, 2.02)
Running/cycling/swimming	–0.11 (–0.61, 0.38)	–0.26 (–0.76, 0.24)	Strength +/– stretching	–0.67 (–2.39, 1.01)	–0.84 (–2.54, 0.85)						
Strength +/– stretching	–0.85 (–2.54, 0.88)	–0.83 (–2.55, 0.89)	Walking + exercise	–0.27 (–0.98, 0.45)	–0.58 (–1.24, 0.08)						
Walking + exercise	–0.440 (–1.100, 0.221)	–0.581 (–1.239, 0.076)									
ASM/BMI											
Physically inactive	1.00 (Reference)	1.00 (Reference)	Walking	1.00 (Reference)	1.00 (Reference)	Running/cycling/swimming	1.00 (Reference)	1.00 (Reference)	Strength +/– stretching	1.00 (Reference)	1.00 (Reference)
Walking	0.03 (0.02, 0.05)	0.04 (0.02, 0.05)	Running/cycling/swimming	0.01 (–0.01, 0.03)	0.01 (–0.02, 0.03)	Walking + exercise	–0.00 (–0.04, 0.03)	–0.01 (–0.04, 0.03)	Walking + exercise	0.04 (–0.06, 0.14)	0.01 (–0.09, 0.11)
Running/cycling/swimming	0.05 (0.03, 0.07)	0.04 (0.03, 0.06)	Strength +/– stretching	–0.03 (–0.10, 0.04)	–0.03 (–0.09, 0.04)						
Strength +/– stretching	0.01 (–0.06, 0.07)	0.00 (–0.06, 0.07)	Walking + exercise	0.01 (–0.02, 0.04)	0.04 (0.02, 0.07)						
Walking + exercise	0.04 (0.02, 0.07)	0.04 (0.02, 0.07)									

Models were adjusted for age, smoking habits, and healthy diet. Gray-shadowed cells denote statistical significance. Abbreviations: 5STS, five-time sit-to-stand; ASM, appendicular skeletal muscle mass; BMI, body mass index; CI, confidence interval.

Table S2. Associations of physical activity habits with physical performance measures and estimated appendicular skeletal muscle mass in female participants (n = 2222).

	**Univariate β (95% CI)**	**Adjusted (95% CI)**		**Univariate β (95% CI)**	**Adjusted (95% CI)**		**Univariate β (95% CI)**	**Adjusted (95% CI)**		**Univariate β (95% CI)**	**Adjusted (95% CI)**
Handgrip strength/BMI											
Physically inactive	1.00 (Reference)	1.00 (Reference)	Walking	1.00 (Reference)	1.00 (Reference)	Running/cycling/swimming	1.00 (Reference)	1.00 (Reference)	Strength +/– stretching	1.00 (Reference)	1.00 (Reference)
Walking	0.05 (0.02, 0.08)	0.04 (0.01, 0.07)	Running/cycling/swimming	0.01 (–0.03, 0.059)	0.096 (0.046, 0.146)	Walking + exercise	0.07 (–0.01, 0.15)	0.07 (–0.01, 0.14)	Walking + exercise	0.12 (–0.08, 0.31)	0.09 (–0.11, 0.281)
Running/cycling/swimming	0.07 (0.02, 0.11)	0.05 (0.01, 0.09)	Strength +/– stretching	0.10 (–0.02, 0.219)	0.06 (–0.08, 0.19)						
Strength +/– stretching	0.15 (0.02, 0.27)	0.15 (0.03, 0.27)	Walking + exercise	0.12 (0.06, 0.18)	0.17 (0.10, 0.24)						
Walking + exercise	0.17 (0.12, 0.23)	0.14 (0.09, 0.20)									
Relative muscle power											
Physically inactive	1.00 (Reference)	1.00 (Reference)	Walking	1.00 (Reference)	1.00 (Reference)	Running/cycling/swimming	1.00 (Reference)	1.00 (Reference)	Strength +/– stretching	1.00 (Reference)	1.00 (Reference)
Walking	0.31 (0.19, 0.42)	0.25 (0.14, 0.36)	Running/cycling/swimming	0.28 (0.10, 0.46)	0.33 (0.19, 0.46)	Walking + exercise	0.09 (–0.11, 0.30)	0.08 (–0.12, 0.28)	Walking + exercise	0.11 (–0.33, 0.54)	0.08 (–0.35, 0.51)
Running/cycling/swimming	0.58 (0.42, 0.75)	0.49 (0.33, 0.64)	Strength +/– stretching	0.31 (–0.13, 0.75)	0.35 (–0.02, 0.72)						
Strength +/– stretching	0.61 (0.51, 1.007)	0.57 (0.14, 1.000)	Walking + exercise	0.45 (0.23, 0.67)	0.41 (0.24, 0.58)						
Walking + exercise	0.75 (0.51, 0.97)	0.62 (0.41, 0.82)									
Allometric muscle power											
Physically inactive	1.00 (Reference)	1.00 (Reference)	Walking	1.00 (Reference)	1.00 (Reference)	Running/cycling/swimming	1.00 (Reference)	1.00 (Reference)	Strength +/– stretching	1.00 (Reference)	1.00 (Reference)
Walking	3.02 (0.00, 6.03)	1.66 (–1.12, 4.54)	Running/cycling/swimming	8.00 (3.16, 12.75)	9.46 (5.55, 13.37)	Walking + exercise	–0.68 (–6.18, 4.83)	–9.99 (–6.44, 4.45)	Walking + exercise	0.05 (–11.56, 11.64)	–0.68 (–12.22, 10.87)
Running/cycling/swimming	10.97 (6.56, 15.39)	7.39 (2.68, 12.09)	Strength +/– stretching	6.79 (–5.12, 18.70)	9.55 (–1.20, 20.28)						
Strength +/– stretching	9.80 (–2.73, 22.34)	7.45 (–4.28, 19.19)	Walking + exercise	9.40 (3.41, 15.39)	8.57 (3.73, 13.42)						
Walking + exercise	12.41 (6.45, 18.37)	8.92 (3.25, 14.55)									

Specific muscle power											
Physically inactive	1.00 (Reference)	1.00 (Reference)	Walking	1.00 (Reference)	1.00 (Reference)	Running/cycling/swimming	1.00 (Reference)	1.00 (Reference)	Strength +/– stretching	1.00 (Reference)	1.00 (Reference)
Walking	0.97 (0.29, 1.65)	1.66 (–1.22, 4.54)	Running/cycling/swimming	1.56 (0.29, 2.83)	0.92 (0.20, 1.65)	Walking + exercise	–0.25 (–1.29, 0.79)	–0.31 (–1.35, 0.73)	Walking + exercise	–0.93 (–2.87, 1.001)	–0.93 (–2.83, 0.97)
Running/cycling/swimming	2.53 (1.52, 3.54)	8.59 (4.41, 12.77)	Strength +/– stretching	1.44 (–1.48, 4.36)	1.75 (–0.12, 3.63)						
Strength +/– stretching	2.41 (–0.32, 5.15)	8.81 (–2.99, 20.62)	Walking + exercise	1.34 (–0.10, 2.79)	–0.58 (–0.26, 1.43)						
Walking + exercise	2.32 (1.002, 3.32)	1.77 (0.48, 3.06)									
Calf circumference											
Physically inactive	1.00 (Reference)	1.00 (Reference)	Walking	1.00 (Reference)	1.00 (Reference)	Running/cycling/swimming	1.00 (Reference)	1.00 (Reference)	Strength +/– stretching	1.00 (Reference)	1.00 (Reference)
Walking	–0.26 (–0.67, 0.16)	–0.32 (–0.73, 0.10)	Running/cycling/swimming	–0.01 (–0.60, 0.58)	0.14 (–0.28, 0.56)	Walking + exercise	–0.04 (–0.60, 0.51)	–0.05 (–0.59, 0.49)	Walking + exercise	0.475 (–0.80, 1.79)	0.21 (–1.001, 1.44)
Running/cycling/swimming	–0.27 (–0.85, 0.32)	–0.38 (–0.97, 0.20)	Strength +/– stretching	–0.03 (–1.63, 1.569)	–0.32 (–1.54, 0.896)						
Strength +/– stretching	–0.29 (–2.04, 1.47)	–0.33 (–2.07, 1.41)	Walking + exercise	0.20 (–0.59, 0.99)	0.09 (–0.45, 0.64)						
Walking + exercise	–0.06 (–0.88, 0.77)	–0.19 (–1.002, 0.64)									
ASM/BMI											
Physically inactive	1.00 (Reference)	1.00 (Reference)	Walking	1.00 (Reference)	1.00 (Reference)	Running/cycling/swimming	1.00 (Reference)	1.00 (Reference)	Strength +/– stretching	1.00 (Reference)	1.00 (Reference)
Walking	0.03 (0.01, 0.04)	0.02 (0.01, 0.03)	Running/cycling/swimming	–0.00 (–0.02, 0.02)	0.04 (0.01, 0.06)	Walking + exercise	0.02 (–0.02, 0.05)	0.02 (–0.02, 0.05)	Walking + exercise	0.06 (–0.02, 0.14)	0.05 (–0.03, 0.13)
Running/cycling/swimming	0.02 (0.01, 0.04)	–0.00 (–0.02, 0.02)	Strength +/– stretching	0.01 (–0.04, 0.06)	–0.01 (–0.08, 0.06)						
Strength +/– stretching	0.03 (–0.02, 0.08)	0.01 (–0.04, 0.06)	Walking + exercise	0.02 (–0.01, 0.04)	0.06 (0.03, 0.09)						
Walking + exercise	0.04 (0.02, 0.07)	0.04 (0.01, 0.09)									

Models were adjusted for age, smoking habits, and healthy diet. Gray-shadowed cells denote statistical significance. Abbreviations: 5STS, five-time sit-to-stand; ASM, appendicular skeletal muscle mass; BMI, body mass index; CI, confidence interval.
